# Dynamical system modeling in schizophrenia: a narrative review of computational psychiatry frameworks

**DOI:** 10.1080/19585969.2026.2724875

**Published:** 2026-09-06

**Authors:** Héléne Soydemir, Christophe Gauld, Mathieu Desroches, Baptiste Pignon, Damien Depannemaecker

**Affiliations:** a Aix Marseille Univ, INSERM, INS, Inst Neurosci Syst, Marseille, France; b SANPSY, CNRS, UMR 6033, Service de psychopathologie du développement, Hospices Civils de Lyon, Bordeaux, France; c Inria Branch at the University of Montpellier, Montpellier, France; d Basque Center for Applied Mathematics, Bilbao, Spain; e APHP, Paris, France

**Keywords:** Schizophrenia, dynamical systems modeling, symptom trajectories, translational research, precision psychiatry, computational psychiatry

## Abstract

The temporal evolution of schizophrenia symptoms is inherently complex and non-linear. Clinical states fluctuate between relatively stable and highly disorganised phases. These dynamics are only partially captured by conventional diagnostic frameworks. This limitation has motivated the development of models that explicitly account for temporal structure and state transitions. Dynamical systems theory provides a rigorous framework for describing non-linear interactions and evolving symptom trajectories. Recent advances in longitudinal data acquisition and computational analysis have enabled the identification of informative dynamical features and the characterisation of patient-specific trajectories. This narrative review synthesises the growing body of work applying dynamical systems theory to schizophrenia spectrum disorders. It distinguishes theoretical models, data-driven approaches, and frameworks empirically evaluated against real patient data. It also examines methodological limitations, translational challenges, and the need for further longitudinal validation. The aim is to provide a coherent conceptual framework for understanding schizophrenia as a dynamic process, to promote cross-disciplinary dialogue, and to guide future research in translational computational psychiatry.

## Introduction

Schizophrenia (SZ) is a severe and chronic psychiatric disorder that profoundly disrupts brain function and daily life. It is widely recognised as a neurodevelopmental disorder that typically begins in early adulthood. With a lifetime prevalence of approximately 0.7%, it affects around 21 million people worldwide, and is a major contributor to global disability-adjusted life years (McGrath et al. 2008; Vigo et al. 2016). SZ is characterised by a heterogeneous constellation of symptoms, including hallucinations, delusions, cognitive deficits, disorganised thinking, and motivational impairments (Jiang et al. 2025). Symptom profiles vary markedly across individuals and may evolve throughout the course of illness (Austin et al. 2015).

Yet, the diagnosis and management of schizophrenia still rely on static categorical classifications (DSM and ICD). These symptom-based categories are defined by clinical consensus rather than underlying neurobiology. They align poorly with findings from neuroscience and genetics, have limited value for predicting treatment response, and fail to capture the fundamental mechanisms underlying the disorder (Insel et al. 2010). Moreover, cultural context can influence symptom expression and clinical interpretation, further limiting the universal applicability of purely symptom-based classifications. Misdiagnosis affects up to 25% of patients, largely due to the reliance on subjective clinical judgement, resulting in suboptimal treatment (Ayano et al. 2021). Given that longer durations of untreated psychosis are associated with poorer outcomes, early detection and timely intervention are critical (Salazar de Pablo et al. 2024).

Approximately 20%–30% of patients exhibit resistance to conventional antipsychotic treatments, experiencing persistent symptoms and significant side effects without adequate therapeutic benefit (Siskind et al. 2017; Chand et al. 2020). Treatment resistance is also associated with elevated rates of psychiatric comorbidity, reflecting overlapping symptom dimensions rather than clearly delineated disease entities (Pizzol et al. 2023). This high level of multimorbidity underscores the aetiological complexity of SZ, arising from the intricate interplay of biological, environmental, and psychological factors. These observations highlight the need for more flexible and temporally informed diagnostic frameworks, particularly when addressing psychiatric comorbidities (Szoke et al. 2024).

### Dynamics in schizophrenia

Since Kraepelin’s early description of *dementia praecox* as a chronic degenerative illness (Kraepelin 1913), SZ has been viewed as progressive mental decline. Subsequently, Bleuler introduced the term SZ to describe a fundamental *‘splitting’* of psychic functions,affect, thought, and behaviour, reflecting global mental disorganisation (Bleuler 1911). He emphasised core structural features, proposing the *‘four As’* (affective flattening, associative loosening, ambivalence, autism), recognising both clinical heterogeneity and possible dynamic stability.

Focusing on patients’ subjective experience, Clérambault described *‘mental automatism’,* a sequence of passive, anideic phenomena lacking clear ideational content (Clérambault 1920). His emphasis on the temporal unfolding of psychopathological phenomena anticipated later attempts to interpret symptom evolution in terms of underlying neural dynamics (Ey 1954). Concurrently, advances in neurophysiology transformed the understanding of brain function. Early work by du Bois-Reymond and Lapicque established neurons as excitable elements generating electrical signals, laying the foundation for the study of how neuronal dynamics produce time-dependent activity patterns (Finger 2001).

Today, SZ is understood as a dynamic interplay of symptoms, whose combinations and severity fluctuate over time and across individuals (Schneider 1959). Symptom dynamics involve intermittent changes and complex temporal dependencies, reflecting interactions between multiple processes and potential transitions between distinct clinical states (Tschacher et al. 1996, 1997; Grassberger 1986). Long-term outcome studies, including World Health Organisation(WHO) data (Harrison et al. 2001), highlight substantial heterogeneity in illness trajectories: some individuals achieve sustained recovery, whereas others experience relapsing courses or persistent severe impairment (Häfner et al. 1999). This variability may reflect neurodevelopmental processes in which early vulnerabilities interact with postnatal events, including altered apoptosis and synaptic pruning, shaping diverse clinical trajectories without major histopathological signs characteristic of a classical neurodegenerative process (Woods 1998).

SZ is often characterised by an insidious onset, with symptoms emerging gradually rather than through an abrupt transition (Fusar-Poli et al. 2013). The prodromal phase may involve subtle self-disturbances, such as hyper-reflexivity, thought interference, and altered perception–imagination boundaries, reflecting changes within the psychosis spectrum (van Os 2014). The basic symptoms paradigm further focused on subjective anomalies, including derealization and diminished self-evidence, as markers of vulnerability to psychosis (Schultze-Lutter et al. 2007).

The Ultra High Risk(UHR) concept was developed to identify individuals at elevated risk of psychosis based on clinical markers and risk profiles (McGorry et al. 2014). This approach led to assessment tools such as Comprehensive Assessment of At Risk Mental States, which assesses attenuated symptoms (CAARMS), which evaluates attenuated psychotic symptoms, Brief Limited Intermittent Psychotic Symptoms, part of UHR criteria (BLIPS), and genetic risk combined with functional decline (Yung et al. 2005). However, UHR criteria capture only a subset of individuals who later develop psychosis, and transition rates remain relatively low (approximately 15–30% over two years). Moreover, intensive monitoring raises concerns regarding resource demands, false positives, stigma, and limited individual-level predictive accuracy (Fusar-Poli et al. 2012). These limitations have motivated the development of dynamic, individualised, and transdiagnostic risk models (Van Os and Guloksuz 2017).

Clinical staging models were introduced to characterise illness trajectories and improve early detection, particularly among young individuals at risk for severe mental disorders (McGorry et al. 2014), with empirical validity for predicting clinical outcomes (Salokangas 2008). SZ is often conceptualised across three broad phases—premorbid, prodromal, and chronic—associated with distinct clinical, cognitive, and neurobiological profiles (Ciompi 1989; Agius et al. 2010). These observations have motivated multimodal and cross-diagnostic staging approaches that integrate dimensional symptoms with neurobiological measures (Shah et al. 2020).

However, understanding illness progression requires distinguishing between different levels of temporal organisation. *Neural dynamics* refer to time-dependent changes in neuronal activity across brain networks, whereas *clinical symptom dynamics* describe temporal variations in the intensity and combination of positive, negative, and disorganised symptoms across individuals. These levels are related but not identical: similar neural activity patterns may lead to different symptom trajectories due to environmental influences, internal states, and individual variability, while similar clinical manifestations may emerge from distinct neural configurations. Consequently, although staging frameworks often assume an ordered progression through illness stages (McGorry et al. 2014), SZ typically follows heterogeneous and non-linear trajectories characterised by fluctuations, remissions, and relapses that challenge static classification and prediction (Ciompi 1997; van Os and Kapur 2009).

SZ is viewed as a multifactorial and dynamic disorder shaped by complex interactions between genetic and environmental factors (Rantala et al. 2022). Psychotic dimensions may share common developmental pathways, including early psychosocial stressors that contribute to latent vulnerability. Transdiagnostic effects of such stressors have been observed across subclinical symptom domains, supporting the existence of shared risk mechanisms (Pignon et al. 2021). Genomic studies further reveal pleiotropic variants across multiple psychiatric disorders that converge on shared molecular networks (Lee et al. 2025), supporting a dimensional and dynamic model of disease emergence and progression.

### Dynamical systems theory: A primer for psychiatric applications

SZ has accordingly been conceptualised as a “dynamical disease”, in which qualitatively distinct symptomatic trajectories emerge from a common nonlinear generative process rather than from a single, homogeneous aetiology (Glass and Mackey 1988; Schiepek et al. 1992). This perspective laid the conceptual groundwork for modern investigations of dynamical approaches to SZ (West 2012; Bosl et al. 2025). Dynamical Systems Theory(DST)-based approaches offer a way to connect biological, cognitive, and clinical levels by focusing on the temporal structure and underlying mechanisms of symptom evolution. They support a non-linear psychiatry aimed at modelling and predicting psychopathological transitions (West 2012).

DST originates from classical mechanics and the mathematical description of systems evolving continuously over time through differential equations (Newton et al. 1999). It also draws on Leibniz’s development of calculus, which provided a systematic way to describe continuous change, namely, how small moment-to-moment variations can accumulate into large-scale transformations. Poincaré introduced a qualitative approach to nonlinear dynamics, focusing on the geometric organisation of trajectories in phase space rather than on explicit analytical solutions (Poincaré 1893).

The full set of possible configurations constitutes the phase space (here, ‘phase’ refers not to oscillatory phase but more generally to all configurations the system can explore), where each point corresponds to a unique system state (Nolte 2010; Perko 2013; Strogatz 2024). The phase space is defined by the system’s state variables, which may represent biological, cognitive, or clinical quantities, including membrane potentials, synaptic currents, or symptom dimensions in SZ applications. Their temporal evolution is governed by underlying (bio)physical laws and model parameters, such as synaptic weights, symptom interaction strengths, treatment effects, or Nernst potentials. The equations define a flow in phase space, with trajectories tracing the system’s temporal evolution (Breakspear 2017; Rabinovich et al. 2006).

**Figure 1.**
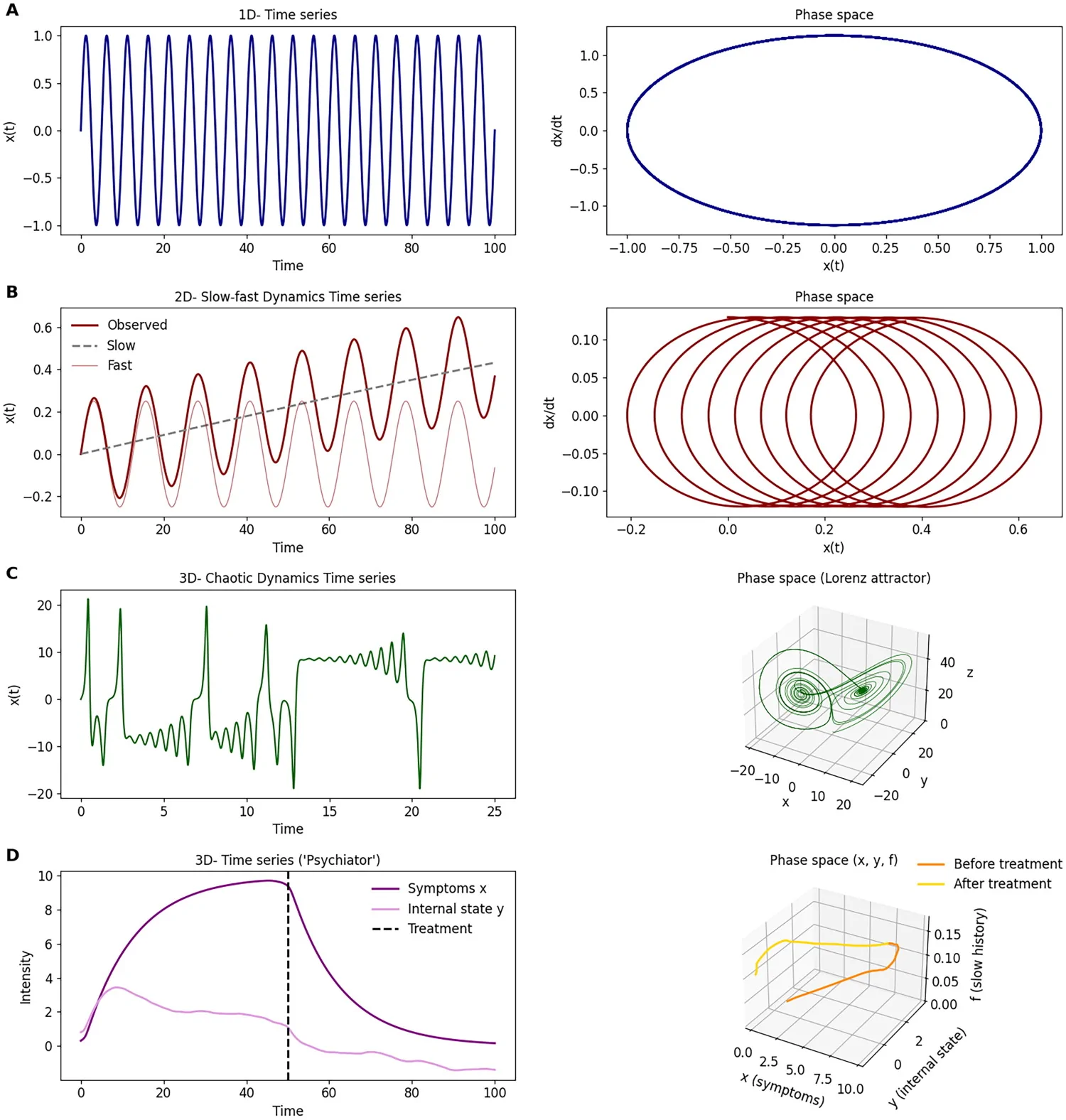
Conceptual trajectories illustrating dynamics of 1D, 2D, and 3D systems. Panel A (blue): 1D sine wave time series. Shows x(t) over time. Panel B (red): 2D simplified model of neural-like dynamics, showing Time series with slow (dashed) and fast components. Panel C (green): 3D Lorenz system. Left: time series of x(t). Right: Lorenz attractor in 3D. Panel D: 3D ‘Psychiator Toy-model’ (Gauld and Depannemaecker, 2023a, 2023b). Left: time series of symptoms x and internal state y, with treatment indicated. Right: phase space (x, y, and f) before (orange) and after (green) treatment.

Over time, these trajectories often converge towards stable geometric structures, or attractors, which characterise the system’s long-term behaviour. Conceptual examples of trajectories for 1D, 2D, and 3D systems are shown in Figure 1, illustrating how time series translate into geometric structures in phase space, and how interventions can alter system dynamics.

A fixed point represents a stable state in which the state variables remain constant over time; a limit cycle corresponds to a persistent oscillatory pattern generated by the system itself; and a strange attractor reflects aperiodic, chaotic dynamics that remain bounded within phase space without ever exactly repeating. A system is structurally stable when small parameter changes leave its qualitative behaviour unchanged, producing only minor modifications of its attractors. In contrast, a bifurcations, or so-called phase transition, occurs when a small parameter variation triggers a qualitative shift in the system’s dynamics, leading to a new dynamical regime (Izhikevich 2007; Breakspear 2017).

Non-linear brain dynamics have been proposed to contribute to the cognitive and perceptual disturbances observed in SZ (An der Heiden 2006; Breakspear 2017). Psychotic states may emerge from nonlinear interactions between affective and cognitive processes, where small changes in underlying parameters can trigger bifurcations (Ciompi 1997; Izhikevich 2007). Separately, within a given regime, nonlinear systems can also exhibit sensitive dependence on initial conditions, whereby infinitesimally close states diverge over time, a phenomenon known as the ‘butterfly effect’ (Lorenz 1963). Both properties illustrate how disproportionate consequences can follow from comparatively small causes in nonlinear dynamical systems. Bifurcations may further involve a form of hysteresis, whereby prior states shape future trajectories and produce persistent changes once a critical point is crossed.

**Figure 2.**
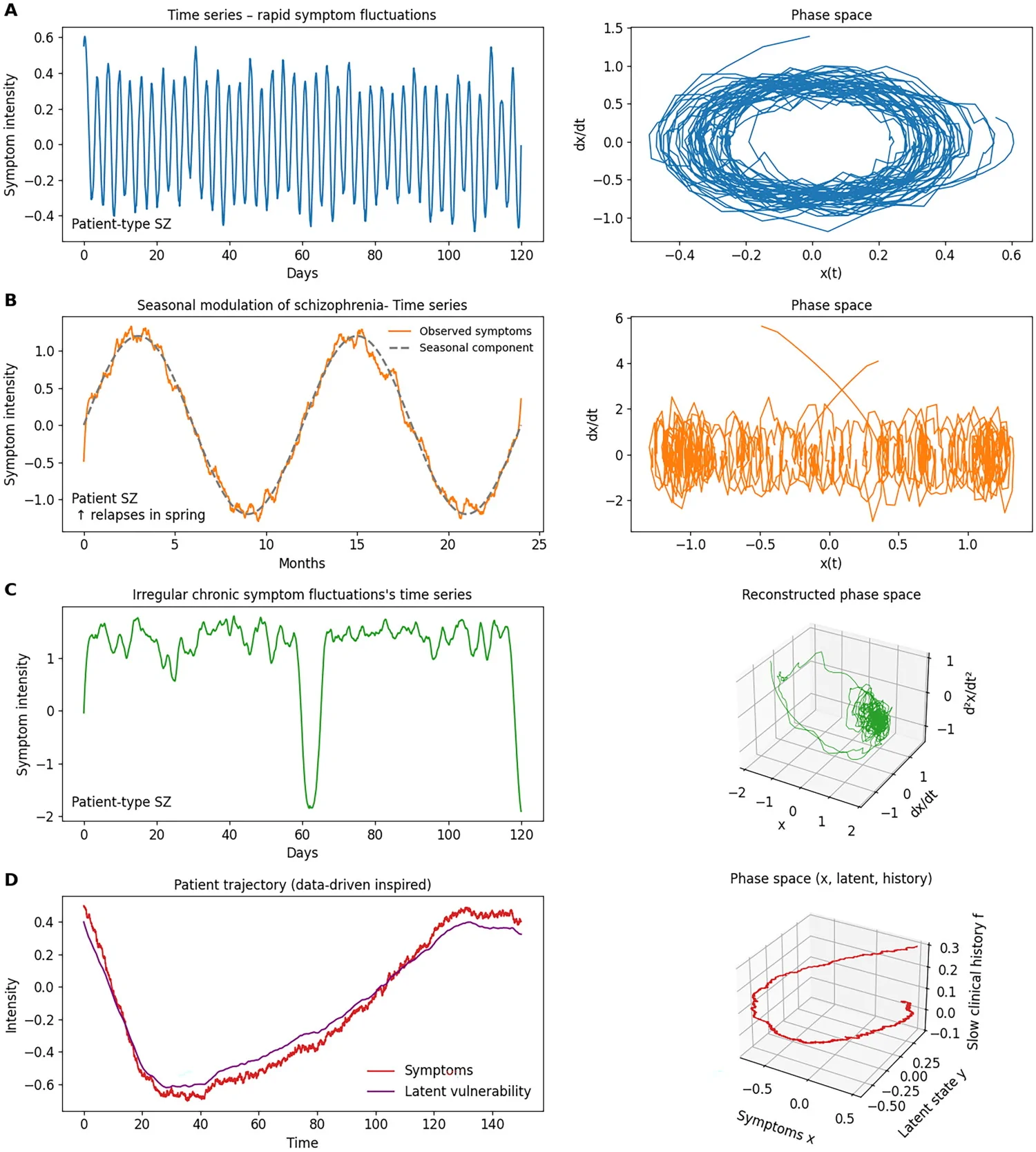
Patient-derived trajectories illustrating symptom dynamics and phase space. All data are synthetic, simulated to reproduce dynamics observed in real patients. Panel A (blue): Rapid daily symptom fluctuations. Left: time series of smoothed symptoms. Right: phase space (x vs dx/dt). Panel B (orange): Seasonal modulation of SZ symptoms. Left: time series with slow seasonal component (dashed) and fast noise. Right: phase space (x vs dx/dt). Shows increased relapses in spring. Panel C (green): Irregular chronic fluctuations (chaos-like). Left: time series. Right: reconstructed 3D phase space (x, dx/dt, d²x/dt²). Panel D (red): Data-driven latent clinical dynamics. Left: time series of symptoms and latent vulnerability. Right: 3D phase space (x = symptoms, y = latent state, f = slow clinical history). (1) Conceptual discrete-time neural network inspired by DST, using phase-space representations of *S*(*t*) and hallucination-related outputs. (2) Discrete-time analogues of ODEs, capturing coupled fast–slow dynamics. (3) Implicit discrete-time formulation, conceptually related to a stochastic differential equation. (4) Conceptual DST-based framework addressing affect–cognition interactions and qualitative bifurcation concepts.

Together, these mechanisms provide a framework for understanding the high variability of SZ trajectories, where gradual fluctuations in emotional and cognitive states can give rise to markedly different clinical courses across individuals (Scrimali 2018). This heterogeneity is well documented empirically, with long-term follow-up studies reporting highly divergent outcome trajectories among patients with comparable initial presentations (Huber et al. 1980; Hegarty et al. 1994; Austin et al. 2015). Diverse dynamical patterns, including rapid daily fluctuations, seasonal modulations, irregular chronic fluctuations, and latent clinical dynamics, are illustrated by the patient-derived (synthetic) SZ symptom trajectories shown in Figure 2.

### Self-Organisation, collective dynamics, and their breakdown in psychopathology

The DST approach conceptualises behaviour as an emergent property of self-organising systems, in which stable behavioural patterns arise from nonlinear interactions among neural, bodily, and environmental processes rather than from centralised control (Huys et al. 2014). Complexity theory emerged formally in the mid-twentieth century through pioneering work on nonlinear dynamical systems and deterministic chaos (Lorenz 1963). Here, chaos does not mean randomness or disorder, but a regime in which the system remains deterministic while becoming highly sensitive to internal states and external perturbations.

Complex systems with many interacting elements can exhibit emergent collective dynamics irreducible to individual components. This phenomenon is formalised in Haken’s theory of Synergetics (Haken 1977). According to this framework, systems driven far from equilibrium like the brain, can spontaneously self-organize, producing coordinated patterns of activity without the need for a central ‘conductor’ or controller. In this process, a small number of slow variables, or ‘order parameters’, dominate the collective dynamics, while the many faster, microscopic processes adapt to these dominant modes. This aligns with the principle of ‘disorder creates order’ advocated by Prigogine (Prigogine and Nicolis 1977).

Despite the high dimensionality of neural systems, brain activity often evolves along a small number of collective variables, effectively collapsing onto a low-dimensional manifolds (Langdon et al. 2023). In other words, the brain’s dynamics behave as if constrained to a smaller space of stable patterns. These patterns are not arbitrary. They reflect forms of organisation that maximise structured information while respecting the brain’s physical and functional constraints (Haken 2006). Disruptions of these organising structures can distort continuous attractors into discrete or unstable states, impairing the representation of internal variables and potentially contributing to cognitive dysfunction and psychopathology. Pathological conditions may reflect either an inability to reach such organised regimes or an excessive sensitivity to fluctuations that destabilises functional patterns (Kelso 1995; Haken 2006, 2013; van der Maas 2024).

### Schizophrenia as a nonlinear dynamical system

Over subsequent decades, this interdisciplinary framework expanded to encompass self-organisation in biological and social systems (Haken 1990), as well as applications to cognition and psychology (Kelso 1995; Scheffer et al. 2024). Such nonequilibrium dynamics support cognitive flexibility, multistability, and rapid reconfiguration of functional networks, while also increasing sensitivity to perturbations (Nartallo-Kaluarachchi et al. 2025; Deco et al. 2017). Thus, brain function is a continuously evolving dynamical process (Jirsa 2020). A useful analogy is that of a tennis player preparing to return a serve: readiness emerges from ongoing coordinated motion, enabling rapid adaptation with minimal energy expenditure.

Together, these principles provide a framework for conceptualising SZ as a disorder of nonlinear brain dynamics, arising from impaired self-organisation and altered collective dynamics (Kelso 1995; Haken 2013; Scheffer et al. 2024). Consistent with this view, electroencephalogram (EEG) studies have shown that irregular oscillations and increased temporal variability correlate with cognitive and clinical states in SZ (Itil 1977; West 2012). This dynamical perspective also aligns with the neurodevelopmental hypothesis, whereby environmental and psychosocial factors acting during critical periods shape disease trajectories and individual risk (Szoke et al. 2020).

Within this dynamical framework, neuromodulators such as dopamine (DA), serotonin, and acetylcholine can be understood as control parameters that reshape the geometry of the underlying manifold and thereby alter the trajectories accessible to brain dynamics. Neural mass model studies illustrate this principle: increased dopaminergic input expands the repertoire of dynamical patterns and shifts their characteristic frequencies, highlighting the role of neuromodulation in regulating brain activity (Depannemaecker et al. 2025).

These mechanistic insights have motivated recent applications of DST to precision psychiatry. Latent neurodynamical features extracted from routine electrophysiological recordings have been proposed to enable personalised trajectory monitoring (Bosl et al. 2025). Similarly, individualised “virtual brain” models aim to simulate patient-specific brain dynamics and support personalised treatment decisions (Preti et al. 2026).

The aim of this narrative review is to examine how dynamical systems theory has been applied to characterise, explain, and model SZ. It integrates theoretical frameworks, empirical evidence, and emerging computational approaches, distinguishing established dynamical principles from exploratory hypotheses, and evaluates how DST-based models contribute to a more mechanistic understanding of SZ pathophysiology. To this end, we review dynamical models of SZ at both brain and symptom levels, alongside the empirical and methodological foundations needed for clinical translation.

## Towards a DST modeling of schizophrenia

Early efforts to model brain- and symptom-level dynamics in psychiatric disorders, including SZ and depression, illustrated the broader applicability of DST to psychopathology (King et al. 1984; Ciompi 1997; Huber et al. 1999). Complementing purely data-driven approaches, theory-driven models formalise mechanistic hypotheses and use simulations to investigate how interactions between system components generate observed dynamics. They provide a framework for testing causal mechanisms, generating predictions, and exploring scenarios that cannot be directly examined experimentally (Huys et al. 2016). In computational psychiatry, biophysical modelling and simulation are particularly valuable for linking biological mechanisms to emergent clinical phenomena and for evaluating potential effects of interventions (Montague et al. 2012).

### Methods consideration

This work was conducted in accordance with SANRA guidelines (Baethge et al. 2019), ensuring transparency in article selection and synthesis. Search terms combined SZ with concepts from DST, nonlinear dynamics, attractors, and bifurcations. Article selection prioritised conceptual relevance, historical significance, and methodological contribution, rather than exhaustiveness (1800–2025). Articles in Table 1 were preferentially selected if they employed Ordinary Differential Equations (ODEs) to model dynamic systems, whether or not they were originally validated against empirical data, reflecting a methodological focus on mechanistic modelling.

**Table 1.** Non-exhaustive overview of different approaches using dynamical systems models applied to SZ.

Reference	Model	ODEs	Methods	Variables	Timescale	Dynamics	Data type
**Brain-level models**							
King and Barchas; Huberman (1984)	Nigrostriatal dopaminergic system	✓	DDE (ODEs with delay) + nonlinear iterative map: Simulations, spectral and Lyapunov	Firing rate, DA concentrations, synaptic stores, postsynaptic efficacy	ms–s	Multistability, Period-doubling bifurcations, Chaotic transitions, and Erratic fluctuations linked to Symptom variability	Simulation
McGlashan and Hoffman (2000)	Develop-mentally Reduced Synaptic Connectivity [1]	×	Neural network simulation of speech perception with synaptic pruning	S(t) = synaptic density, Cognitive performance (identified words), Simulated hallucinations (misidentified words)	months-years	Synaptic loss: <30% optimal → 35–40% cognitive decline → >40% hallucinations → plateau	Simulation
An der Heiden (2006)	Recurrent inhibitory cortical circuit	✓	Simulation of delay differential equations, bifurcation analysis	*E*(*t*), *I*(*t*); pyramidal cell output; inhibitory cell output; receptor parameters	ms-s	Periodic and chaotic firing; bifurcations *via* inhibitory receptor changes (DA); healthy → pathological transitions.	Simulation
Loh et al. (2007)	Recurrent attractor model	✓	IF neurons: simulations with Poisson inputs and MF of attractor states	Firing rates of selective pools + inhibitory pool; synaptic currents (AMPA, NMDA, GABA)	ms-s	Stability of spontaneous/persistent attractors; distractibility; attractor transitions and flow; NMDA/GABA modulation effects.	Simulation
Rolls and Deco (2021)	Stochastic recurrent cortical network IF (SDE)	✓	Simulation, attractor stability, exploration of reduced NMDA and GABA effects	Membrane potential, firing rates, NMDA/AMPA/GABA synaptic currents, mean activity of neuron pools	ms-s	Spontaneous (low-firing) and persistent (high-firing) attractor states, noise-driven transitions and destabilisation of cortical activity, conceptually linked to negative and positive symptom dimensions.	Simulation + empirical support
Sun et al. (2023)	BNM (Wong-Wang + Wilson-Cowan)	✓	Coupled ODEs (exc./inh. populations linked by SC), RSFC, energy gaps	Synaptic activity; inputs; global coupling, local parameters	ms-s	Stable fixed points (spirals/damped oscillations) with possible transitions (bifurcation-like barriers)	Empirical
**Symptom-level models**							
Huberman (1987)	Deterministic model of smooth pursuit eye movements	✓	Forced and damped nonlinear oscillator; Bifurcation, power spectra, Poincaré maps, noise effects	Eye deviation angle, Angular velocity	ms-s	Dynamic regimes: stable tracking, asymmetric oscillations, chaotic/unpredictable behaviour, history-dependent transitions, effect of noise on bifurcations	Simulation
Schiepek et al. (1992)	Mixed feedback model of SZ [2]	×	Discrete nonlinear difference equations, Slow-fast dynamics Simulation, logistic map, Feigenbaum route, random fluctuations	Fast: cognitive, stress, withdrawal, emotion, delusions; Slow: cognition, neurochemistry, social skills	days–weeks	Asymptotic fixed points, periodic oscillations, nonlinear feedbacks, episodic patterns, chaotic behaviour	Simulation
Ciompi (1976)	Affect-logic [4]	×	Conceptual dynamic framework	Affect, cognition, psychosocial, biological factors	days-months	Premorbid vulnerability, Decompensation → psychosis, Long-term evolution with attractors, bifurcations, variability in remission and chronicity	Conceptual
Tschacher et al. (1997)	Nonlinear dynamics of psychotic symptoms in SZ [3]	×	Phase space reconstruction, forecasting algorithms, surrogate data testing to distinguish chaos/noise/linearity	Daily rating of psychotic derealization (univariate time series)	days	Three types: (1) nonlinear/chaotic dynamics (57% of patients), (2) linear (AR/ARMA), (3) random/noise.	Empirical
Gauld et al. (2023)	Toy-model ‘Psychiator’	✓	4 coupled nonlinear ODEs; dynamical simulation	Symptoms x, internal factors y, environmental noise z, temporal specificity f	months-years	Nonlinear interactions → multistability, slow–fast dynamics, stochastic perturbations, simulation of treatment effects	Simulation

Note: It included models that apply DST either directly, by formulating systems of ODEs to model symptom dynamics in SZ, or conceptually, by invoking DST concepts without specifying a system of ODEs.

### Brain-level models

Early applications of DST to the central dopaminergic system modelled nigrostriatal dopamine (DA) dynamics as a nonlinear difference equation derived from a delay-differential system. In this model, a parameter *k* governs the efficacy of DA at the postsynaptic receptor. Numerical simulations showed that increasing *k* drives the system through a cascade of period-doubling bifurcations into chaotic firing regimes (King et al. 1984). DA plays a central role in SZ, with receptor overactivity and altered metabolite levels correlating with symptom severity and psychotic episodes (Davis et al. 1991; van Os and Kapur 2009). Together, these findings suggest that the intrinsic nonlinear properties of the dopaminergic system may give rise to irregular neural dynamics, potentially contributing to the heightened behavioural variability observed in SZ, even in the absence of external perturbations (Schiepek 2003; Tschacher et al. 1996, 1997).

A second, complementary route to dynamical instability arises from structural alterations in connectivity rather than from intrinsic dynamics alone. Developmentally reduced synaptic connectivity and progressive pruning degrade global network function, producing hallucination-like errors (McGlashan and Hoffman 2000). Disruption of this attractor-based organisation, particularly through excessive pruning of long-range cortical connections, fragments large-scale brain dynamics and disrupts coordinated activity, reshaping the attractor landscape and creating new, pathological stable minima (Hoffman and McGlashan 2001). This offers a mechanistic explanation for persistent hallucinations and delusions under ambiguous inputs.

At the circuit level, imbalances between excitation and inhibition, together with modulatory effects of DA, destabilise cortical attractors, facilitating erratic transitions between network states. Gradual changes in DA function act as a control parameter, influencing network stability and transitions between healthy and pathological states in SZ (An der Heiden 2006). Recurrent excitatory–inhibitory cortical circuits and thalamo-cortico-striatal loops can exhibit irregular or chaotic firing, which can be captured computationally using coupled differential equations for excitatory input, inhibitory postsynaptic potentials, and output firing rates, with DA modulating inhibitory efficacy.

At the synaptic and receptor level, reduced N-Méthyl-D-Aspartate (NMDA) receptor conductance flattens cortical attractor basins, increasing susceptibility to noise and impairing working memory, attention, and firing rates in prefrontal, orbitofrontal, and anterior cingulate cortices (Loh et al. 2007; Rolls 2021). Computational models, including integrate-and-fire and stochastic recurrent networks with DA modulation, show that interactions between excitatory (NMDA) and inhibitory Gamma-aminobutyric acid (GABA) populations determine attractor stability. Destabilisation of high-activity attractors in dorsolateral prefrontal cortex underlies cognitive symptoms, while shallower basins in orbitofrontal and anterior cingulate regions contribute to negative symptoms. Noise-induced transitions in temporal and auditory association networks, exacerbated by GABA hypofunction, may generate positive symptoms such as hallucinations and delusions. DA modulation can partially restore stability: D1 receptor agonists strengthen high-activity states, improving cognitive and negative symptoms, while D2 antagonists stabilise spontaneous states, reducing positive symptoms (Rolls 2021). Empirical support includes reduced dendritic spine density, decreased GABA-mediated inhibition, and altered functional connectivity and temporal variability observed in functional magnetic resonance imaging (fMRI).

Building on these perspectives, a multi-attractor brain network model has been applied to resting-state fMRI data from healthy controls and patients with SZ, bipolar disorder, and ADHD (Attention-Deficit/Hyperactivity Disorder) (Sun et al. 2023). The model is formulated as a system of coupled ODEs describing the dynamics of excitatory and inhibitory neural populations. By computing all stable attractors, a cross-attractor coordination matrix, and energy gaps, the approach links alterations in global coupling and excitation/inhibition (E/I) balance to psychiatric conditions. Findings indicate lower global coupling and smaller energy gaps, particularly in the Default Mode Network (DMN), somatomotor, and visual networks, in patients, along with a higher E/I ratio in SZ compared with ADHD, suggesting that psychiatric disorders involve modifications in the brain’s attractor landscape.

### Symptom-level models

Beyond the level of neural circuits, dynamical systems principles have also been applied directly to the temporal structure of psychiatric symptoms and individual physiological endophenotypes of SZ. Sitting at the boundary between brain-level and symptom-level descriptions, anomalies in smooth pursuit eye movements in SZ can be interpreted using a deterministic nonlinear framework (Huberman 1987). The oculomotor system can be modelled as a nonlinear oscillator with cubic stiffness, also called a Duffing oscillator. Such a system can shift between different patterns of behaviour: stable tracking, where the eyes follow the target smoothly; symmetry breaking, where the movement becomes biased or uneven; chaotic behaviour, characterised by irregular and unpredictable trajectories; and hysteresis, where the system’s response depends on its past history. These dynamics closely reproduce the irregular eye movements observed in patients. This suggests that SZ may involve inherently unstable eye movement control due to the amplification of nonlinear effects.

At the level of overall symptom trajectories, three complementary approaches have modelled SZ as a nonlinear dynamical process. First, purely theoretical simulations show that small changes in social or cognitive parameters can produce divergent SZ trajectories (Schiepek et al. 1992). These discrete-time models represent cognition, stress, social withdrawal, expressed emotion, and delusions as interacting variables with feedback and stochastic fluctuations, revealing stable and unstable illness regimes. They further show how slow changes in psychological, social, or biochemical parameters can trigger abrupt transitions such as relapse or recovery.

Second, a qualitative conceptual framework, affect-logic, describes schizophrenic psychoses as dissipative structures, whereby complex symptom dynamics emerge from nonlinear interactions among biological, cognitive, and social processes operating far from equilibrium (Ciompi 1997). Despite ongoing perturbations, these interactions generate self-organised patterns and attractors that characterise the disorder’s long-term course. Affective states act as control parameters that modulate cognitive-affective interactions and shape accessible dynamical states (Ciompi 1976), with emergent patterns, attractors, and bifurcations reflecting multistability. Depending on initial conditions and affective modulation, symptom severity may worsen, fluctuate episodically, stabilise, or remit, illustrating how clinical courses of SZ emerge from a common underlying dynamical system influenced by affect and stochastic perturbations.

Third, and unlike the two theoretical accounts above, empirical nonlinear analyses of real symptom time series lend direct support to this picture. Short- and long-term symptom fluctuations analysed with methods such as the Grassberger–Procaccia algorithm and the Sugihara–May approach reveal low-dimensional chaotic attractors, indicating that psychotic dynamics are governed by fractal, self-organising processes rather than random noise (Ciompi 1994). Extending this chaos-theoretic view of SZ, analyses of daily symptom time series reveal distinct dynamical classes, including chaotic, linear autoregressive, or stochastic regimes, and indicate that many patients follow nonlinear, potentially chaotic trajectories (Tschacher et al. 1992, 1996, 1997). Although short clinical time series cannot prove deterministic chaos, features such as decaying predictability and bifurcation-like transitions suggest that SZ dynamics are structured but highly sensitive to small changes.

Finally, a 3 + 1 dimensional toy-model using a non-linear DST framework has been developed to describe the nonlinear progression of psychiatric symptoms (Gauld and Depannemaecker 2023b). Variables include symptoms (x), internal factors (y), environmental noise (z), and temporal specificity (f). The model simulates different clinical scenarios: healthy, episodic, kindling-like, and highly environment-sensitive disorders, and can test treatment effects. This framework explicitly links symptom dynamics to the concept of SZ as a dynamical disease: rather than being static, symptom profiles emerge from interactions between intrinsic neuronal or cognitive factors and extrinsic environmental influences, evolving over time in complex, sometimes abrupt ways. By mapping these trajectories in phase space, the model illustrates how transitions between clinical states (e.g. remission, relapse, or exacerbation) correspond to shifts in attractor landscapes, providing a mechanistic perspective on the temporal variability and unpredictability of SZ. While theoretical, it offers a concrete tool for predictive, personalised, and intervention-oriented computational psychiatry, bridging the gap between longitudinal symptom observations and mechanistic models.

### Data in schizophrenia

Until recently, the study of SZ has been limited by the scarcity of high-quality longitudinal datasets. Early reviews already highlighted the ‘extreme shortcoming of longitudinal research’ in the field (Häfner et al. 1999), a limitation that largely persists today. Ciompi emphasised both decades-long and day-to-day variability in symptom expression (Aebi and Ciompi 1989), illustrating the need for data capturing within-person variations across micro (daily) and macro (yearly) timescales (Ciompi 1989; Nelson et al. 2017). Such temporal richness is necessary to trace how genetic vulnerability and environmental context interact to shape symptom onset and course.

The development of Ecological Momentary Assessment (EMA) has opened new avenues for understanding psychiatric disorders through the lens of DST, enabling extraction of real-time symptom dynamics and within-subject variability often hidden in cross-sectional assessments (Misdrahi et al. 2023; Gauld et al. 2025; Barbosa et al. 2025). For example, in Substance Use Disorders(SUD), optimised linear SARIMAX models characterising bidirectional interactions between cues and craving were replicated by two DST models describing escalation and saturation dynamics (Gauld et al. 2025).

Beyond SZ, the WARN-D project illustrates what such infrastructures could look like at scale, combining intensive longitudinal monitoring *via* wearables with an open machine learning competition to crowdsource prediction of depression onset (Fried et al. 2023), exemplifying an open-science approach to model validation that could similarly benefit DST-based research in SZ. More recently, analyses on FACE-SZ have revealed robust clinical predictors of relapse (Barbosa et al. 2025), medication adherence (Misdrahi et al. 2023), and quality of life, using statistical and machine learning tools such as Cox models, random forests, and classification trees. However, these focus on point-in-time predictions rather than the latent structural and temporal dynamics of symptom trajectories (Barbosa et al. 2025).

### Model validation and model personalisation

By fitting model parameters to observed data, model inversion can allows DST to accurately capture individual variability in brain or symptom dynamics. This process not only tests the model’s validity against empirical observations but also tailors the model to reflect personalised trajectories, enabling more precise diagnosis and intervention (Ziaeemehr et al. 2025).

Beyond parameter fitting, personalisation within a DST framework has recently been reframed as a control engineering problem, in which psychopathology is understood as a distortion of the geometry of low-dimensional cognitive–affective manifolds rather than as an isolated biological dysfunction. Clinical interventions can then be modelled as control inputs that reshape the manifold itself to restore healthy dynamics, and closed-loop, N-of-1 experimental paradigms combining dense longitudinal measurements with strategically designed perturbations have been proposed to train individualised surrogate models of a person’s manifold, supporting the simulation of counterfactual interventions to guide personalised treatment design (Bosl et al. 2025). In a related translational direction, dynamical features extracted from routine electrophysiological recordings have been proposed as a practical, scalable substrate for personalised trajectory monitoring, offering a lightweight complement to more computationally intensive manifold-based approaches (Bosl et al. 2025).

## Discussion

The use of dynamical systems models and complexity theory in the study of psychological and psychiatric phenomena significantly enriches behavioural analysis. The models examined here—ranging from brain-level neuronal systems (King et al. 1984; Loh et al. 2007; Sun et al. 2023) to symptom-level dynamics (Ciompi 1976; Huberman 1987; Schiepek et al. 1992; Gauld and Depannemaecker 2023b, a) provide a framework for understanding emergent processes, multistability, bifurcations, and chaotic or erratic transitions within complex systems. These properties, observable across different temporal and spatial scales, can complement classical statistical approaches in psychology, such as linear regression or ANOVA.

At the brain level, these models illustrate how short-term dynamics, chaos, and erratic fluctuations, can relate to observable behaviours or symptoms, such as variability in motor, perceptual, or cognitive responses. More recent models, such as Sun et al.(2023), combine excitatory and inhibitory populations coupled through structural brain connectivity to study stability and state transitions, highlighting the relevance of multilayer approaches for capturing interactions between local and global scales (Sun et al. 2023).

At the symptom level, different works presented in this narrative review demonstrate the value of dynamical modelling for understanding nonlinear symptom trajectories, the occurrence of crises or decompensations, and responses to interventions (Ciompi 1976; Schiepek et al. 1992; Gauld and Depannemaecker 2023b, 2023a). Models incorporating fast–slow dynamics, multistability, and stochastic perturbations offer a conceptual approach to link internal processes (cognitive, emotional) and environmental factors to clinical outcomes. Clinically, this view implies that symptoms are not entirely random and that interventions can exploit the system’s short-term determinism, targeting chaotic regimes to stabilise favourable behaviour (Elbert et al. 1992; Tschacher et al. 1997). Overall, a parsimonious nonlinear framework may better explain SZ than traditional multicausal models, with implications for both theory and therapeutic strategies.

At the individual level, idiographic DST frameworks and EMA approaches allow reconstruction of patient-specific dynamic landscapes, identifying potential Early Warning Signals(EWS) for clinical transitions (Schiepek 2003; Friston et al. 2012; Van de Leemput et al. 2014; Schreuder et al. 2020, 2023). In psychosis specifically, as a system’s resilience diminishes, even a minor perturbation may be sufficient to push it past a tipping point (Feyaerts et al. 2026). Approaching such a critical transition is typically marked by critical slowing down, a reduced speed of return to equilibrium following perturbation. This is reflected in increasing variance and autocorrelation, offering a candidate EWS of impending clinical change (Feyaerts et al. 2026). While conceptually compelling, the practical utility of these EWS are constrained by measurement noise, partial observability, and high inter-individual heterogeneity (Nelson et al. 2017; Barbosa et al. 2025).

Other approaches such as Dynamic Causal Modelling, energy landscape analyses, network control theory, and whole-brain simulations have revealed how alterations in effective connectivity, synaptic gain, and functional state stability may underlie cognitive inflexibility and symptom maintenance (Fogelson et al. 2014; Braun et al. 2021; Li et al. 2023; Tyras and Wojnar 2025). While informative for studying general neural dynamics, these approaches remain primarily theoretical and are not tailored to the explicit modelling of clinical symptoms, which constrains their use for translational applications.

Conversely, recurrent neural networks and reservoir computing capture nonlinear temporal dependencies in neural and behavioural time series, enabling predictive and discriminative analyses, though these remain largely data-driven and non-mechanistic (Yan et al. 2019). These limitations are increasingly documented in SZ-specific machine learning research: models achieving strong classification performance often remain interpretability-limited “black boxes” that provide clinicians with little mechanistic justification for their outputs (Vázquez et al. 2021), and broader challenges related to data heterogeneity, external validation, and clinical implementation continue to constrain their translation into psychiatric practice (Idowu et al. 2025).

Bridging these two extremes, mechanistic concepts such as slow-fast dynamics, bifurcations, and critical transitions offer a theoretical link between neural dynamics and observable symptom trajectories, and may help explain symptom volatility, abrupt state changes, and multi-timescale variability in psychiatric disorders (Rabinovich et al. 2006; Pavlidis et al. 2024).

However, these approaches face broader methodological limitations. Clear links between phenomenological descriptors and dynamical classifications remain difficult to establish (Tschacher et al. 1997), and implementing coupled or multilayer models remains technically challenging. Moreover, intensive longitudinal data collection introduces practical and ethical constraints. Clinical implementation is limited by the burden of frequent assessments, unequal access to digital tools, and ethical concerns regarding the prediction of psychotic episodes. Intensive monitoring also raises issues related to informed consent, data privacy, transparency of predictive models, and the assumption of stable environments and reliable symptom reporting across diverse cultural, socioeconomic, and healthcare contexts (Mabire-Yon et al. 2025).

Even existing longitudinal studies face structural and logistical challenges. Standardised, scalable infrastructures for multimodal data collection are still lacking, which hinders reproducibility, and long-term follow-up is further complicated by high dropout rates, heterogeneous clinical courses, and sparse or irregular sampling (Hegarty et al. 1994). Translating DST concepts into clinically actionable tools will therefore require high-frequency, multimodal, longitudinal datasets, ideally from multi-centre cohorts, to test mechanistic hypotheses and confirm predictive markers before routine application can be considered. Thus, DST currently serves more as a theoretical and mechanistic framework than a fully validated clinical instrument.

## Conclusion

Despite these limitations, DST provides a mechanistic framework for integrating neural dynamics, symptom trajectories, and environmental influences across multiple temporal scales. Rather than relying on static diagnostic categories, it formalises attractors, bifurcations, multistability, and regime shifts to explain the heterogeneous and non-linear evolution of schizophrenia (Breakspear 2017; Mabire-Yon et al. 2025). Realising the translational potential of DST will require rigorous validation, longitudinal multimodal datasets, and dynamical markers in real-world patient populations (Barbosa et al. 2025; Leboyer and Llorca 2025). Equally important is the development of context-sensitive models that account for cultural, socioeconomic, and healthcare-system variability influencing symptom expression, monitoring, and care pathways.

Finally, it is important to remember that dynamical modelling is not intended to perfectly replicate reality, but rather to provide useful approximations of the underlying mechanisms driving behaviour. Although simplified, such models are valuable for identifying key dynamical properties, exploring regime shifts, and generating mechanistic hypotheses without requiring full biological realism. Explaining cognition requires intermediate theoretical descriptions that bridge neural activity and behaviour, since neural correlations alone do not provide a mechanistic understanding (Marr 2010; Krakauer et al. 2017).

While studies of neural implementation are necessary for testing causal hypotheses, the challenge lies in articulating why a purely reductionist approach may be insufficient. In this sense, the limitations of models are not a weakness but a prerequisite for understanding complexity—a principle that continues to guide the careful application of complex models in mental health research and psychiatry (Epstein 2008; Smaldino 2016).

## Glossary

**bifurcations** A qualitative change in the dynamics of a system caused by a small change in a parameter, leading to the emergence or disappearance of attractors. Bifurcations can correspond to transitions between stable symptom states in computational models of psychiatric disorders. 7

**Grassberger–Procaccia algorithm A** nonlinear time-series analysis method used to estimate the correlationdimension of an attractor from scalar observations, allowing the identification of low-dimensional deterministic chaos in empirical data by assessing how the number of state-space point pairs scales with distance. 17

**manifold** A lower-dimensional surface embedded in phase space that organises the dynamics of a system. In neural and psychiatric models, manifolds can capture correlated patterns of brain activity or constrain the evolution of symptom trajectories. 11

**Neural mass model** Neural mass models are mathematical models that describe the collective dynamics of large populations of neurons by averaging their activity into a small number of state variables. They are commonly used to model mesoscale cortical dynamics and to account for macroscopic electrical and magnetic signals measured with electroencephalography (EEG) and magnetoencephalography (MEG). 12

**phase space** An abstract space in which all possible states of a system are represented, with each axis corresponding to a state variable. Trajectories in phase space describe how the system evolves over time. 7

**state variable** A variable representing the current condition or state of a system at a given time, e.g. neural activity, symptom severity, or network connectivity. State variables evolve over time according to the system’s dynamics and are used in modelling and simulation of psychiatric disorders. 7

**Sugihara–May method** A nonlinear forecasting approach that tests for state-dependent dynamics by reconstructing the system’s attractor from time-series data and evaluating predictive skill, thereby distinguishing deterministic nonlinear processes from stochastic noise. 17

## Supplementary Material

sanra.pdf

Glossary_suppmat.pdf

## Abbreviations:

BLIPS: Brief Limited Intermittent Psychotic Symptoms, part of UHR criteria. 5
CAARMS: Comprehensive Assessment of At Risk Mental States, which assesses attenuated symptoms. 5
DST: Dynamical Systems Theory. 6, 14, 17, 20–22
EMA: Ecological Momentary Assessment. 21, 23
EWS: Early Warning Signals. 23, 24
ODEs: Ordinary Differential Equations. 14, 16, 20
SARIMAX: Seasonal Auto-Regressive Integrated Moving Average with exogenous variables. 21
SUD: Substance Use Disorders. 21
SZ: Schizophrenia. 2–7, 9, 12–21, 24
UHR: Ultra High Risk. 5
WHO: World Health Organisation. 4
