# Supplementary material for: Dynamical system modeling in schizophrenia: a narrative review of computational psychiatry frameworks
Source: Dialogues Clin Neurosci. 2026 Sep 6;28(1):327–42. doi: 10.1080/19585969.2026.2724875 (PMC13552316; doi:10.1080/19585969.2026.2724875)
Supplement: Supplementary file 1 [file TDCN_A_2724875_SM9929.pdf]

Supplementary Materials : Glossary of Terms and Acronyms

Glossary

attractor A stable state or set of states toward which a dynamical system evolves over time. In neuroscience and psychiatry, attractors can represent stable patterns of brain activity or symptom configurations in disorders like schizophrenia. 1

bifurcations A qualitative change in the dynamics of a system caused by a small change in a parameter, leading to the emergence or disappearance of attractors. Bifurcations can correspond to transitions between stable symptom states in computational models of psychiatric disorders. 1

chaotic systems Chaotic systems are deterministic nonlinear dynamical systems that exhibit sensitive dependence on initial conditions, leading to exponential divergence of nearby trajectories in phase space. Despite being governed by deterministic equations, their long-term behavior is effectively unpredictable. Chaotic dynamics are characterized by aperiodicity, positive Lyapunov exponents, and complex attractor structures, and they occur in a wide range of physical, biological, and computational systems. 1

critical transition A sudden and often irreversible shift from one stable state to another in a dynamical system, typically preceded by early-warning signals such as critical slowing down. In psychiatry, this may correspond to abrupt changes in symptom severity or disease state. 1

Grassberger–Procaccia algorithm A nonlinear time-series analysis method used to estimate the correlation dimension of an attractor from scalar observations, allowing the identification of low-dimensional deterministic chaos in empirical data by assessing how the number of state-space point pairs scales with distance. 1

irreversibility A process that produces entropy and cannot be reversed without altering the system or its surroundings, reflecting broken time-reversal symmetry in nonequilibrium systems. Then it's a time-asymmetric process in which neural activity evolves with dissipation and entropy production, preventing exact reversal of past states. In brain dynamics, irreversibility reflects nonequilibrium operation, information processing, and directional interactions across neural systems. 1

manifold A lower-dimensional surface embedded in phase space that organizes the dynamics of a system. In neural and psychiatric models, manifolds can capture correlated patterns of brain activity or constrain the evolution of symptom trajectories. 1

Neural mass model Neural mass models are mathematical models that describe the collective dynamics of large populations of neurons by averaging their activity into a small number of state variables. They are commonly used to model mesoscale cortical dynamics and to account for macroscopic electrical and magnetic signals measured with electroencephalography (EEG) and magnetoencephalography (MEG). 1

Ordinary Differential Equation (ODEs) Ordinary Differential Equation; a mathematical equation describing the rate of change of a variable over time as a function of its current state, commonly used to model dynamic systems such as brain activity or symptom trajectories in psychiatry.. 1

phase space An abstract space in which all possible states of a system are represented, with each axis corresponding to a state variable. Trajectories in phase space describe how the system evolves over time. 1

phase Transitions A transition between distinct dynamical regimes of a system, occurring when gradual parameter changes lead to abrupt qualitative shifts in attractor structure, stability, or variability. A sudden reorganization of neural or behavioral dynamics whereby small changes in control variables (e.g., neuromodulation, stress, connectivity) lead to abrupt shifts between functional states. In psychopathology, such transitions may correspond to symptom onset, remission, or relapse. 1

self-organization Process by which coherent spatiotemporal structure or functional order emerges spontaneously from local interactions among system components, without external control or centralized coordination. Self-organization typically occurs in open, nonequilibrium systems where energy or information flows sustain dynamics, and small fluctuations can be amplified through nonlinear interactions. The resulting macroscopic patterns are distributed, robust, and adaptive, often exhibiting multistability and sensitivity to parameter changes. Property of neural systems whereby coherent activity patterns and functional networks emerge from local neuronal interactions, driven by nonequilibrium dynamics, synaptic plasticity, and nonlinear feedback, rather than by centralized control. 1

slow-fast dynamics A property of dynamical systems in which different components evolve on markedly different timescales. Fast variables change quickly, while slow variables evolve more gradually. This separation of timescales can lead to phenomena such as critical transitions, hysteresis, and transient dynamics, and is particularly relevant in neuroscience for understanding neural population activity and state changes. 1

state variable A variable representing the current condition or state of a system at a given time, e.g., neural activity, symptom severity, or network connectivity. State variables evolve over time according to the system's dynamics and are used in modeling and simulation of psychiatric disorders. 1

Sugihara–May method (Empirical Dynamic Modeling) A nonlinear forecasting approach that tests for state-dependent dynamics by reconstructing the system's attractor from time-series data and evaluating predictive skill, thereby distinguishing deterministic nonlinear processes from stochastic noise. 1

Acronyms

BITS Back in the Saddle. 1

BLIPS Brief Limited Intermittent Psychotic Symptoms, part of UHR criteria. 1

CAARMS Comprehensive Assessment of At Risk Mental States, which assesses attenuated symptoms. 1

CSD Critical Slowing Down. 1

DST Dynamical Systems Theory. 1

EMA Ecological Momentary Assessment. 1

EWS Early Warning Signals. 1

ODEs Ordinary Differential Equations. 1

SARIMAX Seasonal Auto-Regressive Integrated Moving Average with exogenous variables. 1

SUD Substance Use Disorders. 1

SZ Schizophrenia. 1

UHR Ultra High Risk. 1

WHO World Health Organization. 1
