# Supplementary material for: Dynamical system modeling in schizophrenia: a narrative review of computational psychiatry frameworks
Source: Dialogues Clin Neurosci. 2026 Sep 6;28(1):327–42. doi: 10.1080/19585969.2026.2724875 (PMC13552316; doi:10.1080/19585969.2026.2724875)
Supplement: Supplementary file 2 [file TDCN_A_2724875_SM9930.pdf]

SANRA Checklist

Section/Topic	Item	What is expected	Reported on Page/Line	Reported on Section/Paragraph
**TITLE**	**1. Title**	The title must clearly indicate that the article is a *narrative review* or *literature review*.	p. 1	Title
**ABSTRACT**	**2. Structured summary**	Structured summary including: **Background and Objective**, **Methods**, **Key Content and Findings**, **Conclusions**.	p. 1-2, l. 14–28	Abstract
**INTRODUCTION**	**3. Background**	Explain why the review was needed and place it in the context of current knowledge.	p. 2–11	Introduction (§"Dynamics in Schizophrenia"; §"From Dynamical Systems to Neuropsychiatry"; §"Self-Organization and Collective Dynamics in Complex Systems"; §"Schizophrenia as a Nonlinear Dynamical System")
	**4. Objectives**	Clearly state the objectives of, or questions addressed by, the review.	p. 12, l. 258-264	End of Introduction (final paragraph: "The aim of this narrative review is to examine how dynamical systems theory has been applied. . . ")
**METHODS**	**5. Literature search**	Describe the search strategy: databases, time period covered, language, inclusion/exclusion criteria, study types, etc.	p. 13–19	Section 2, "Methods Consideration"
**DISCUSSION**	**6. Narrative**	Discuss the main findings from the literature, their limitations (study quality, bias, lack of validation, etc.), and future research perspectives.	p. 21–23	Section "Discussion"
	**7. Summary**	Conclude by interpreting the findings in the context of clinical practice, health policy, or future research.	p. 24	Section "Conclusion"
